# Comparison of gut microbiota in male MAFLD patients with varying liver stiffness

**DOI:** 10.3389/fcimb.2022.873048

**Published:** 2022-08-03

**Authors:** Yuheng Zhang, Su Yan, Shifeng Sheng, Qian Qin, Jingfeng Chen, Weikang Li, Tiantian Li, Xinxin Gao, Lin Wang, Li Ang, Suying Ding

**Affiliations:** ^1^ Health Management Center, The First Affiliated Hospital of Zhengzhou University, Zhengzhou, China; ^2^ Center for Precision Medicine, First Affiliated Hospital of Zhengzhou University, Zhengzhou, China; ^3^ College of Public Health, Zhengzhou University, Zhengzhou, China

**Keywords:** metabolic dysfunction-associated fatty liver disease, liver stiffness, gut microbiota, comparative genomics, whole-genome sequencing

## Abstract

**Purpose:**

In this study, we examined the changes to the composition and function of the gut microbiota from patients with metabolic dysfunction-associated fatty liver disease (MAFLD).We compared patients in a case group (liver stiffness (LSM) ≥ 7.4 kPa) with a matched control group (LSM < 7.4 kPa) and investigated the correlation between characteristics of the microbiota and other biochemical indicators.

**Methods:**

The study looked at a total of 85 men with MAFLD, 17 of whom were in the case group and 68 of whom were in the control group. We measured waist circumference, blood pressure, and body mass index, as well as clinical parameters including liver stiffness, enzyme levels, cholesterol levels, and fat attenuation. Whole-genome shotgun sequencing technology and the MetaCyc database were then used to detect the composition and major pathways of the gut microbiota for each patient. Statistical analyses were performed, including the chi-square test, the student’s t-test, the Wilcoxon rank-sum test, and the Mann–Whitney test.

**Results:**

Whole-genome sequencing showed that the composition of the gut microbiota in patients with an LSM of above 7.4 kPa was significantly different to that of the control group. There were seven bacterial species that were different between the two groups. Prevotella copri, Phascolarctobacterium succinatutens, Eubacterium biforme, and Collinsella aerofaciens were enriched in the case group (P < 0.05). Conversely, Bacteroides coprocola, Bacteroides stercoris and Clostridiales bacterium 1_7_47FAA were decreased in the case group (P < 0.05). Furthermore, after removing low abundance pathways, a total of 32 microbial pathways were found to be significantly different between the two groups. Most pathways enriched in the case group over the control were related to biosynthesis of metabolites including amino acids, vitamins, nucleosides, and nucleotides. Conclusion. The composition and function of the gut microbiota in patients with increased liver stiffness are significantly altered. This observation may provide new avenues to better understand the mechanism of liver fibrosis.

## Introduction

Metabolic dysfunction-associated fatty liver disease (MAFLD) refers to the accumulation of fat in the liver of patients that meets one of the following criteria: obesity, type 2 diabetes, or metabolic dysfunction. MAFLD, formerly known as nonalcoholic fatty liver disease (NAFLD), has a global prevalence of up to 25%, constituting a serious hazard to human health. In view of the increasing incidence of disease and a deeper understanding of the pathogenesis, a panel of international experts renamed NAFLD to MAFLD in 2020 (Eslam et al., 2020). One consequence of MAFLD is the development of liver fibrosis, which can progress to cirrhosis and even liver cancer (Campana and Iredale, 2017). Currently, the only effective treatments for liver fibrosis are the early removal of the underlying cause, or transplantation of the liver. There is convincing evidence from rodent models and human studies showing that if pathogens or injury factors are removed, early liver fibrosis can be reversed (Sun and Kisseleva, 2015; Kisseleva and Brenner, 2021). Recently, a non-invasive technology that can assess the degree of liver fibrosis has been developed, known as FibroTouch. This technique uses a transient elastography measurement to measure liver stiffness (Deng et al., 2016; Xu et al., 2017; Qu et al., 2021; Zhu et al., 2021).

The gut microbiota has recently begun to be considered an important factor in the pathophysiology of many intestinal and extraintestinal diseases, including irritable bowel syndrome, obesity, and diabetes. The liver is the organ with the closest contact with the gut: 75% of its blood supply comes from the portal vein, which contains a large number of bacteria and metabolites absorbed from the gut. Various liver diseases, including alcoholic liver disease, nonalcoholic liver disease, and primary sclerosing cholangitis, are associated with an altered microbiome (Tilg et al., 2016). Furthermore, studies have shown that dysbiosis can increase toxic metabolites, cause liver inflammation and damage, aggravate liver disease, promote disease progression to liver fibrosis, and seriously affect patient outcomes (Beraza, 2019; Zhou et al., 2019; Qin et al., 2021). These studies have predominantly focused on advanced liver fibrosis; however, there are few studies that have analyzed changes in the gut microbiota in the early stages of liver fibrosis. In this study, we aimed to address this question. To examine changes to the gut microbiota during the early stages of liver fibrosis, patients with MAFLD were divided into two groups: those with normal liver stiffness (liver stiffness measurement (LSM) < 7.4 kPa), and those with abnormal liver stiffness (LSM ≥ 7.4 kPa). The LSM increases in tandem with the stiffness of the liver tissue in the detection area (Chinese Medical Association Liver Disease Branch et al., 2019).

## Materials and methods

### Participants and study designverkade

We screened 1770 participants from the physical examination center of the First Affiliated Hospital of Zhengzhou University between March 2018 and May 2019. Inclusion criteria were as follows: (1) male over 18 years old; (2) clinically proven MAFLD according to the Asian Pacific Association for the Study of the Liver guidelines; and (3) legally competent to provide written informed consent. Exclusion criteria were: (1) the diagnosis of other liver diseases except MAFLD (for example hepatitis B or C, autoimmune hepatitis, hemochromatosis, or drug-induced hepatitis); (2) a history of inflammatory bowel disease; (3) treatment with antibiotics, probiotics or proton pump inhibitors within 2 months prior to the study; (4) the presence of an implanted orthopedic steel device or electronic medical device; or (5) the existence of a tumor. All patients provided written informed consent. The study was approved by the ethics committee from the First Affiliated Hospital of Zhengzhou University (2018-KY-56 and 2018-KY-90).

**Figure 1:**
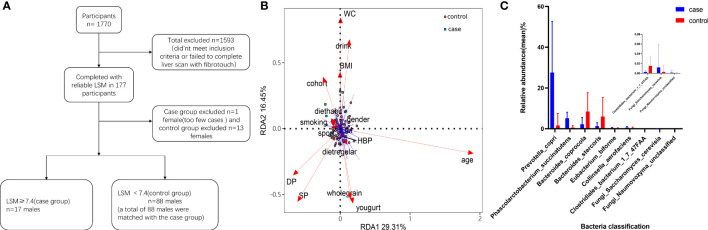
Participant screening process and differential microbial analysis. **(A)** Flow diagram describing participant selection. **(B)** Effects of dietary habits and individual attributes on microflora. **(C)** Species differences between the two groups.

Our study was a retrospective cross-sectional study that was designed to evaluate the gut microbial species in MALFD patients with differing LSM values. A flow diagram describing participant selection is shown in **Figure 1**. After selection, there were 17 patients in the case group and 68 in the control group. The groups were matched by age, gender, waist circumference (WC), body mass index (BMI), dietary habits, and alcohol consumption. Patient characteristics are summarized in **Table 1**.

**Table 1:** Baseline characteristics of the overall cohort.

	LSM<7.4 kPa (N=68)	LSM≥7.4 kPa (N=17)	t/χ2	*P*
Age	47.71 ± 9.87	44.76 ± 10.33	1.089	0.279
WC (cm)	95.27 ± 7.74	96.94 ± 6.54	-0.820	0.415
SBP (mmHg)	131.84 ± 14.45	132.29 ± 14.87	-0.116	0.908
DBP (mmHg)	82.32 ± 10.02	85.47 ± 11.11	-1.134	0.260
BMI (kg/m^2^)	27.36 (26.51~29.05)	28.04 (27.06~29.59)	-1.198	0.318
Regular meals*	N:6;Y:62	N:1;Y:17	/	>0.999
Dietary habit*	MIX:62;MEAT:1;VEGEN:5	MIX:17;MEAT:0;VEGEN:0	/	>0.999
Wholegrains*	N:6;Y:62	N:4;Y:13	/	0.107
Yogurt*	N:6;Y:62	N:4;Y:13	/	0.107
Smoking*	N:58;Y:10	N:15;Y:2	/	>0.999
Drinking*	N:59;Y:9	N:13;Y:4	/	0.283
Sporting	not:2, rarely:60, frequently:6	not:0, rarely:14, frequently:3	1.554	0.460
T2DM*	N:67;Y:1	N:14;Y:3	/	0.024
HP*	N:63;Y:5	N:17;Y:0	/	0.578
WBC (×10^9^/L)	6.29 ± 1.51	6.28 ± 0.97	0.016	0.988
ALT (U/L)	27.50 (20.25~37.75)	40.00 (23.50~55.50)	-2.248	<0.001
AST (U/L)	23.00 (17.25~27.00)	26.00 (22.00~33.50)	-2.379	0.001
GGT (U/L)	34.50 (24.25~59.00)	46.00 (28.50~77.00)	-1.528	0.117
ALB (g/L)	10.75 (8.83~14.46)	11.15 (8.03~13.42)	-0.615	0.753
TBIL (μmol/L)	12.19 ± 5.27	10.70 ± 3.02	1.123	0.265
TC (mmol/L)	74.87 ± 12.06	77.18 ± 13.38	-0.691	0.492
TG (mmol/L)	1.83 (1.36~2.67)	1.85 (1.13~2.93)	-0.044	0.096
HDL (mmol/L)	4.98 ± 1.00	4.98 ± 1.12	-0.017	0.987
LDL (mmol/L)	2.05 ± 1.09	2.09 ± 1.25	-0.135	0.893
FPG (mmol/L)	5.51 (4.90~6.34)	5.41 (5.03~5.94)	-0.312	0.090
HbA1c (%)	5.91 (5.69~6.49)	5.83 (5.61~6.05)	-1.164	0.817
Cr (μmol/L)	72.00 (66.25~83.50)	71.00 (68.50~85.00)	-0.572	0.573
SUA (μmol/L)	6.24 ± 1.00	5.85 ± 0.46	1.316	0.192
CAP (dB/m)	270.14 (253.50~299.89)	300.30 (265.98~326.05)	-1.987	0.031
LSM (kPa)	5.88 ± 1.27	8.94 ± 1.12	-9.049	<0.001

WC, waist circumference; SBP, systolic blood pressure; DBP, diastolic blood pressure; BMI, body mass index; regular meals, Y = regular eating; N = irregular eating; dietary habits (mixed, meat-eating, vegan); yogurt, Y = ate yogurt every day; N = did not eat yogurt every day; smoking, Y =smoked; N = did not smoke; drinking, Y = alcohol consumption; N = no alcohol; sporting, (no exercise, rarely exercise, frequently exercise); T2DM, Type 2 diabetes; HP, hypertension; WBC, white blood cell count; ALT, alanine aminotransferase; AST, aspartate aminotransferase; GGT, gamma-glutamyl transpeptidase; ALB, albumin; TBIL, total bilirubin; TC, total cholesterol; TG, triglycerides; HDL, high-density lipoprotein; LDL, low-density lipoprotein; FPG, fasting plasma glucose; HbA1c, glycosylated hemoglobin; Cre, Creatinine; SUA, Serum uric acid; CAP, fat attenuation value; LSM, liver stiffness.*The fisheries exact probability method is adopted, so there is no statistics.

### Measurement data and laboratory tests

The subjects’ demographic information, behavioral risk factors, weight, height, WC, and blood pressure were collected by trained staff in the First Affiliated Hospital of Zhengzhou University. Samples to measure white blood cells (WBC), alanine transaminase (ALT), aspartate aminotransferase (AST), gamma-glutamyl transpeptidase (GGT), albumin (ALB), total bilirubin (TBIL), total cholesterol (TC), triglycerides (TG), high-density lipoprotein cholesterol (HDL), low-density lipoprotein cholesterol (LDL), fasting plasma glucose (FPG), glycated hemoglobin A1c (HbA1c), creatinine (Cre), and serum uric acid (SUA) were collected from the patients after 12 hours of fasting. On the same day, stool samples were obtained, immediately stored at -20°C, and frozen at -80°C within 30 minutes of collection. The samples were then tested.

### Liver scans

A Toshiba Color Doppler Ultrasound System (APLI0500 TUS-A500) was used to scan patient livers. Abdominal ultrasound diagnosis of fatty liver required two of the following three observations: (1) the near-field echo of the liver was diffusely enhanced, and the echo was stronger than that of the kidney; (2) the structure of the intrahepatic duct was unclear; and (3) the far-field echo of the liver was gradually attenuated. Liver stiffness (LSM) and fat attenuation (CAP) were detected by FibroTouch (FT-C, Wuxi, China). The scans were performed by two experienced doctors.

### DNA extraction and microbiome composition profiling

Genomic DNA extraction from a total of 85 fecal samples was performed using the MagPure Stool DNA KF kit according to the manufacturer’s instructions. DNA libraries were constructed based on DNA nanospheres and shotgun metagenomic sequencing through binding probe anchor synthesis for all samples (MGI2000, MGI, Shenzhen, China). The overall accuracy (≥ 0.8) was used to evaluate the sequencing reads in order to filter out low-quality reads.

The intestinal microbiota was analyzed through classification, annotation, and quantification, based on MetaPhlAn2 with default settings (Truong et al., 2015). This analysis included bacteria, archaea, eukaryotic viruses, and fungi. The number of fungi present was small and is shown in **Supplementary Figure 1**. We then used HUMAnN2 (HMP Unified Metabolic Analysis Network 2) to further generate taxon-specific community function profiles (Franzosa et al., 2018). Neither archaea nor eukaryotic viruses were present in the final dataset; therefore, we did not include these in further analysis.

### Statistical analysis

Statistical analyses were performed using the program R (version 4.0.2; www.r-project.org). Categorical variables are represented by counts, and chi-square tests were used for differential analyses. Continuous variables are expressed as means ± standard deviations or as medians (interquartile ranges). The Kolmogorov–Smirnov test was to test for normality, and Levene’s test was used to test the homogeneity of variances. Their results are presented in **Supplementary Table 1**. Depending on the results, either the Student’s t-test or the Mann–Whitney test were used. *P* < 0.05 was considered statistically significant. Permutational multivariate analysis of variance (PERMANOVA) and redundancy analysis (RDA) were performed to confirm whether liver stiffness was the most important influencing factor. The Shannon index and observed species number (obs) index of each sample was calculated using the vegan package in R. Principal coordinate analysis (PCoA) was performed using ade4 in R for visual analysis. Differences in the microbiota at the genus levels and pathways were analyzed using STAMP (version 2.1.3). Differences between the groups were calculated using the Benjamini–Hochberg false discovery rate (FDR). Species with low occurrences (occurrence rate < 10%) were removed before analyzing the microbiota. Spearman’s correlation analysis was used to analyze the correlations between the patients’ microbiota and other covariates, and the corrplot package was used for visualization.

## Results

### Clinical characteristics

A total of 1770 participants qualified for the study. Of these, 1607 either did not meet the inclusion criteria or failed to complete the liver scan with FibroTouch, and so were excluded. The remaining 163 participants completed the study and their LSM was assessed. Those with an abnormal LSM (LSM ≥ 7.4) were selected for the case group. To form a matched control group, 68 men were selected (all with LSM < 7.4 kPa) from the participants. The final cohort therefore included data from 85 patients (**Table 1**). A flow diagram depicting patient selection is shown in **Figure 1A**. The incidence of type 2 diabetes (T2DM) was higher in the case group than in the control group (*P* < 0.05). The median levels of alanine aminotransferase (ALT) and aspartate aminotransferase (AST) in the case group were 40.00 (interquartile range: 23.50–55.50) U/L and 26.00 (interquartile range: 23.00–33.50) U/L respectively. In both cases, the levels were higher than in the matched control group (*P* < 0.05). The liver stiffness (mean LSM = 8.94 ± 1.12 kPa) and fat attenuation index (median CAP = 300.30 dB/m; interquartile range: 265.98–326.05) were also significantly higher in the case group compared with the control group (*P* < 0.05). The other variables showed no statistically significant differences between the two groups.

### Analysis of factors that influence the gut microbiota

PERMANOVA was used to analyze the basic attributes of the 85 participants (age, WC, systolic blood pressure (SBP), diastolic blood pressure (DBP), BMI, regular diet, dietary habits, consumption of whole grains, consumption of yogurt, smoking, alcohol consumption, exercise level, T2DM, and hypertension (HP)). In both univariate and multivariate analyses, a high LSM had the greatest impact on the gut microbiota of the study participants (*P* < 0.05; **Table 2**).

**Table 2:** Influence of participants’ basic attributes on microbiota composition.

Phenotype	Single factor			Multi-factor		
	F.Model	Variation (R^2^)	*P* (>F)	F.Model	Variation (R2)	*P* (>F)
Cohort	3.913	0.045	0.002	4.046	0.045	0.001
Age	1.128	0.013	0.295	1.333	0.015	0.173
WC	1.128	0.013	0.295	0.775	0.009	0.706
SBP	0.773	0.009	0.720	0.812	0.009	0.632
DBP	0.665	0.008	0.819	0.851	0.009	0.635
BMI	0.842	0.010	0.632	1.650	0.018	0.06
Regular Diet	1.295	0.015	0.200	1.361	0.015	0.155
Dietary habit	0.707	0.008	0.770	0.391	0.004	0.986
Wholegrains	1.770	0.021	0.052	1.283	0.014	0.206
Yogurt	0.888	0.011	0.541	2.066	0.023	0.012
Smoking	1.001	0.012	0.389	1.044	0.012	0.383
Drinking	0.767	0.009	0.720	0.970	0.011	0.439
Sporting	2.739	0.032	0.002	2.676	0.030	0.002
T2DM	1.610	0.019	0.067	0.615	0.007	0.874
HP	0.866	0.010	0.571	1.006	0.011	0.393

We constructed an RDA diagram to examine the relationship between the microbiota and participants’ dietary habits and individual attributes (**Figure 1B**).

### Comparison of gut microbiota

**Figure 2:**
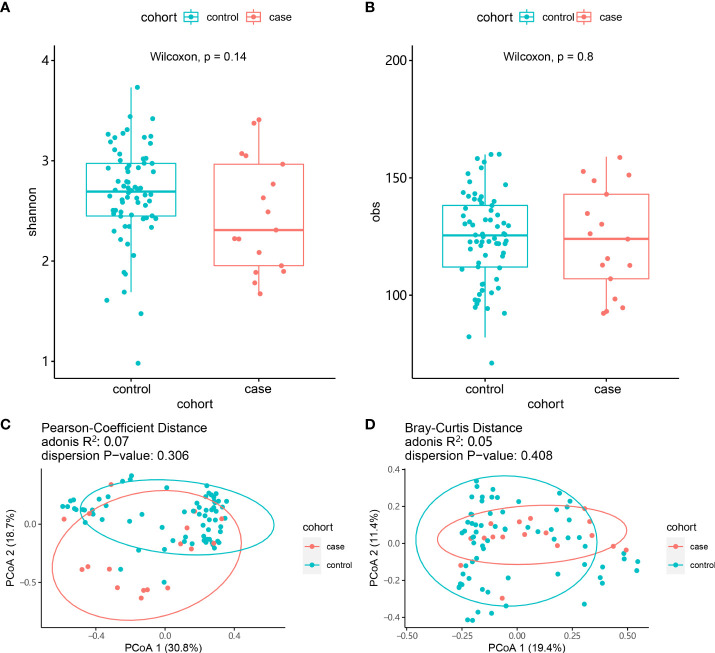
Comparison of the composition of the gut microbiota between the case and control groups. **(A)** α-diversity (Shannon index). **(B)** α-diversity (obs index). **(C)** β-diversity (Bray–Curtis test). **(D)** β-diversity (Pearson test).

We next analyzed the bacteria present in the gut microbiota. The case group was dominated by the phyla Bacteroidetes, Firmicutes and Actinobacteria. In the control group, Bacteroidetes and Firmicutes were dominant. We observed seven bacterial species that differed between the case group and the control group. Furthermore, we observed two fungi (*Naumovozyma unclassified* and *Saccharomyces cerevisiae*) present only in the control group (**Figure 1C**). The bacteria *Prevotella copri, Phascolarctobacterium succinatutens, Eubacterium biforme*, and *Collinsella aerofaciens* were all increased in the case group compared with in the control group (*P* < 0.05). Conversely, *Bacteroides coprocola, Bacteroides stercoris* and *Clostridiales bacterium 1_7_47FAA* were all reduced in the case group (*P* < 0.05). We then assessed the α-diversity of gut microbiota using the Shannon diversity index and the observed species number (obs) index, both of which showed no statistically significant difference between the two groups (*P >* 0.05). To look at β-diversity, we performed principal coordinate analysis (PCoA1) using the Bray–Curtis and the Pearson distance tests. These analyses determined that there was no statistically significant difference between the case and control groups (*P* > 0.05). However, the composition of the gut microbiota from patients in the case group clustered independently compared with patients in the control group (**Figure 2**).

### Correlation analysis of gut microbes and clinical indicators

**Figure 3:**
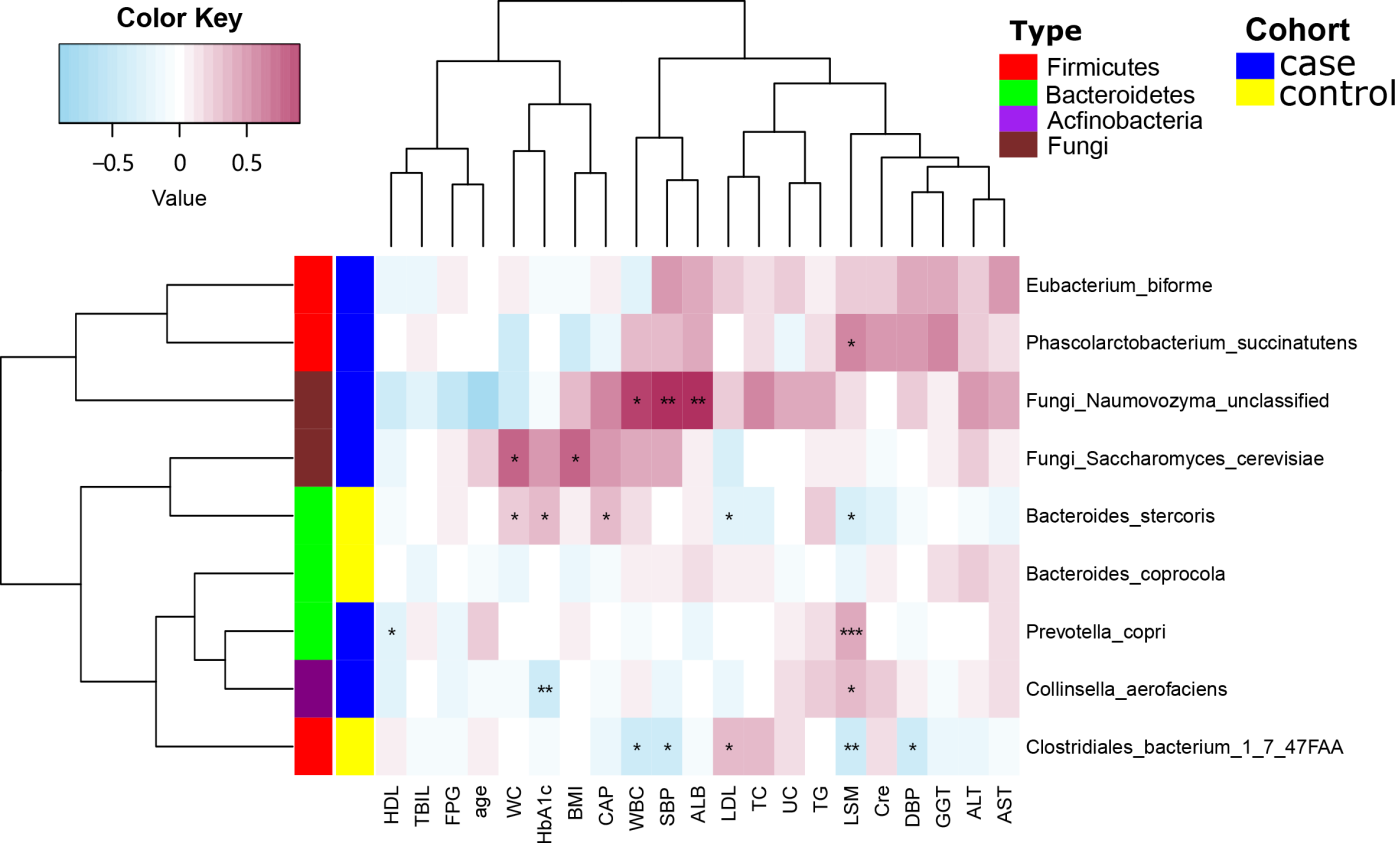
Spearman’s correlation analysis showing the correlations between species abundances and participant characteristics. ^*^ *P* < 0.05, ^**^ *P* < 0.01.

Next, we used Spearman’s correlation analysis to examine the correlation between serological indicators and the abundances of certain species within the gut microbiota. The level of *Phascolarctobacterium succinatutens* was positively correlated with LSM (*P* < 0.05). *Eubacterium biforme* was also positively correlated with LSM (*P* < 0.05), but was inversely correlated with levels of high-density lipoprotein cholesterol (HDL; *P* < 0.01). *Clostridiales bacterium 1_7_47FAA* was negatively correlated with white blood cell count (WBC; *P* < 0.05) but was positively correlated with fasting plasma glucose levels (FPG; *P* < 0.05). *Collinsella aerofaciens* was positively correlated with both LSM and WBC (*P* < 0.05) yet was negatively correlated with HDL (*P* < 0.05). *Bacteroides stercoris* was inversely correlated with low-density lipoprotein cholesterol (LDL) and LSM (*P* < 0.05) but positively correlated with WC, glycated hemoglobin A1c (HbA1c; *P* < 0.05) and CAP (*P* < 0.01). The fungus *Naumovozyma unclassified* was positively correlated with SBP, albumin levels (ALB; *P* < 0.01), and WBC (*P* < 0.05). The fungus *Saccharomyces cerevisiae* was positively correlated with WC and BMI (*P* < 0.05) (**Figure 3**).

### Functional differences in the microbiome of the participants

**Figure 4:**
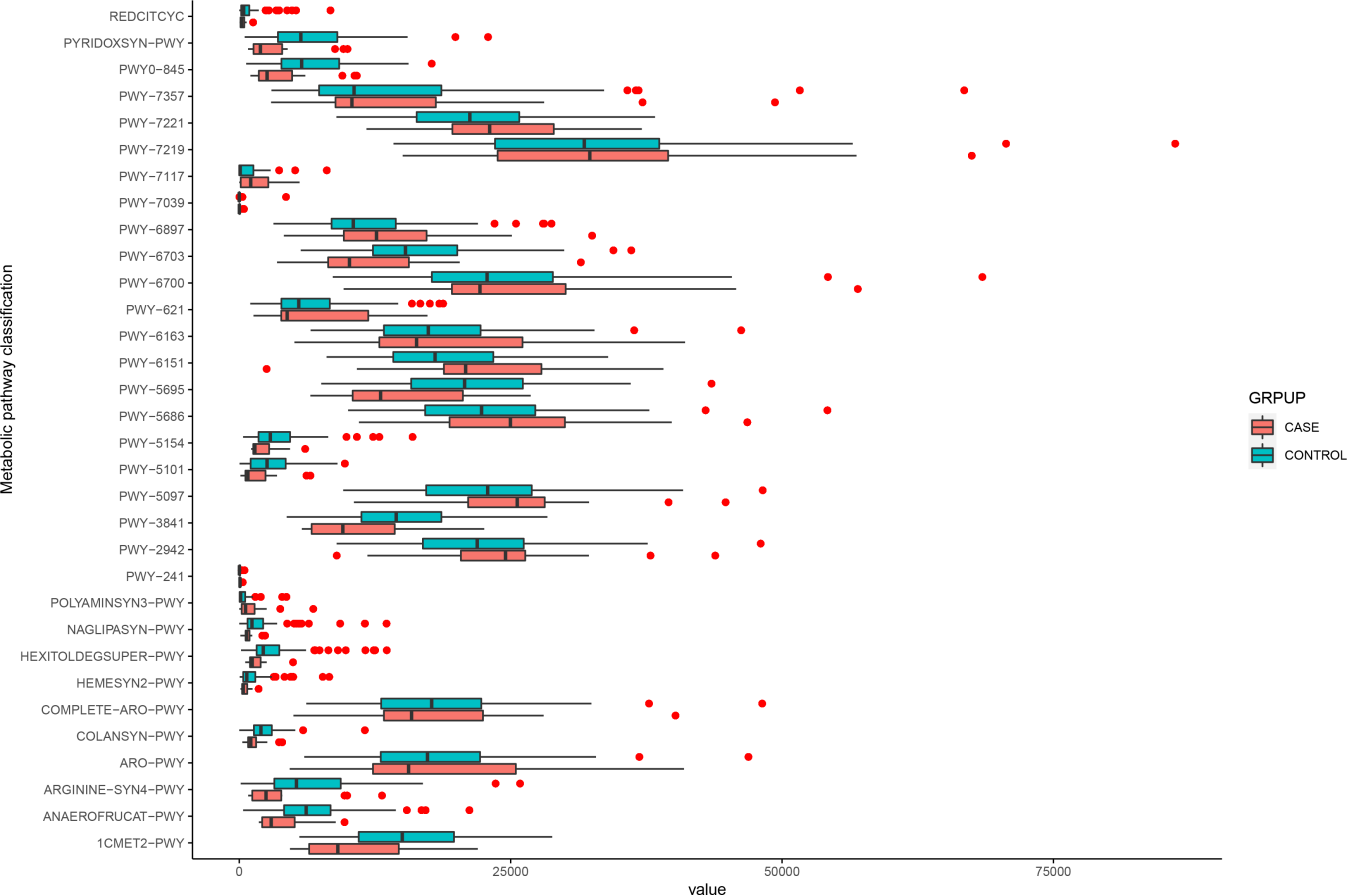
Functional changes in bacterial species between two groups. 32 pathways differed significantly, and 16 were enriched in case group (*P* < 0.05).

We used the MetaCyc database to construct functional profiles for each sample. After removing low abundance pathways, a total of 32 pathways were found to be significantly different between the two groups (**Figure 4**; **Supplementary Table 2**). Of these pathways, 16 were enriched in the case group compared with the control group. Among these, 14 pathways (ARO-PWY, PWY-6163, PWY-5097, PWY-6151, PWY-2942, POLYAMINSYN3-PWY, PWY-6897, PWY-7357, PWY-6700, PWY-5686, PWY-7221, COMPLETE-ARO-PWY, PWY-7039, and PWY-7219) were related to biosynthesis of key metabolites, including amino acids, vitamins, nucleosides, and nucleotides. The remaining two, PWY-7117 and PWY-241, were related to photosynthesis (**Figure 4**).

### Correlation analysis of gut microbes and pathways

**Figure 5:**
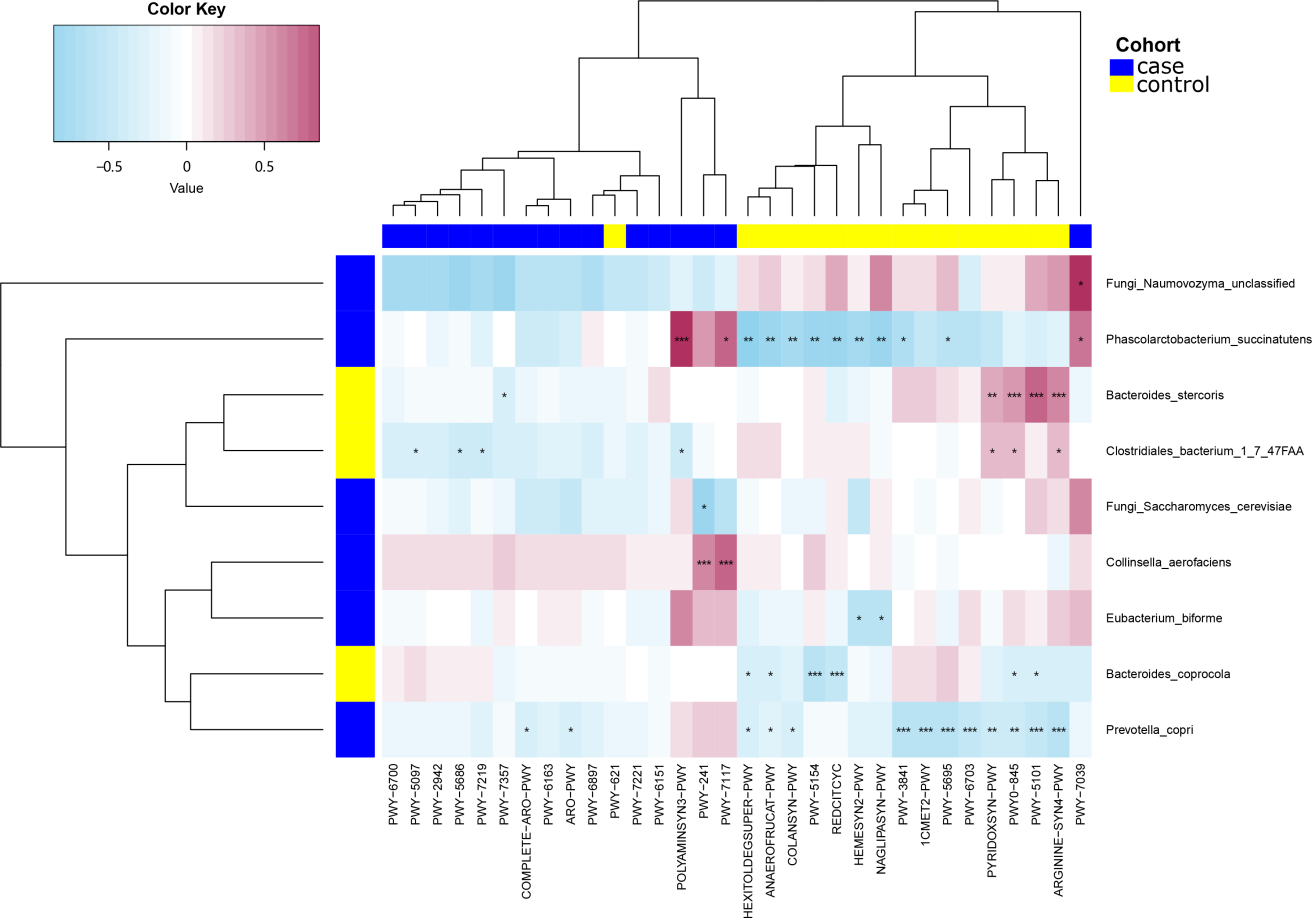
Spearman’s correlation analysis showing the correlations between species abundances and microbial pathways. ^*^ *P* < 0.05, ^**^ *P* < 0.01, ^***^ *P* < 0.001.

We then applied Spearman’s correlation analysis to analyze the correlation between bacterial species and microbial pathways (**Figure 5**). The level of *Clostridiales bacterium 1_7_47FAA* was positively correlated with PWY-5154, PYRIDOXSYN−PWY, PWY0−845, PWY−5101 (*P* < 0.05), and ARGININE−SYN4−PWY (*P* < 0.01). *Bacteroides stercoris* was positively correlated with PWY0−845, ARGININE−SYN4−PWY, PWY−5101 (*P* < 0.001), and PYRIDOXSYN−PWY (*P* < 0.01), but negatively correlated with PWY−7357 (*P* < 0.05). *Collinsella aerofaciens* was positively correlated with PWY−241, PWY−7117 (*P* < 0.001), and POLYAMINSYN3−PWY (*P* < 0.01), yet negatively correlated with PYRIDOXSYN−PWY, PWY0−845 and, ARGININE−SYN4−PWY (*P* < 0.05). *Phascolarctobacterium succinatutens* was positively correlated with POLYAMINSYN3−PWY (*P* < 0.05). *Bacteroides coprocola* was negatively correlated with REDCITCYC, PWY−5154 (*P* < 0.001), HEXITOLDEGSUPER−PWY, ANAEROFRUCAT−PWY, and PWY−7039 (*P* < 0.05). *Eubacterium biforme* was positively correlated with POLYAMINSYN3−PWY (*P* < 0.001), but negatively correlated with NAGLIPASYN−PWY, ANAEROFRUCAT−PWY, PWY−384, and PYRIDOXSYN−PWY (*P* < 0.05). *Prevotella copri* was negatively correlated with PWY−5695 (*P* < 0.001), COLANSYN−PWY, HEXITOLDEGSUPER−PWY, PWY−3841, 1CMET2−PWY, PWY−6703, and PWY−5101 (*P* < 0.05). The fungus *Naumovozyma unclassified* was positively correlated with PWY−7039 (*P* < 0.05). The fungus *Saccharomyces cerevisiae* was negatively correlated with PWY−241 (*P* < 0.05).

### Correlation analysis of pathways and clinical indicators

**Figure 6:**
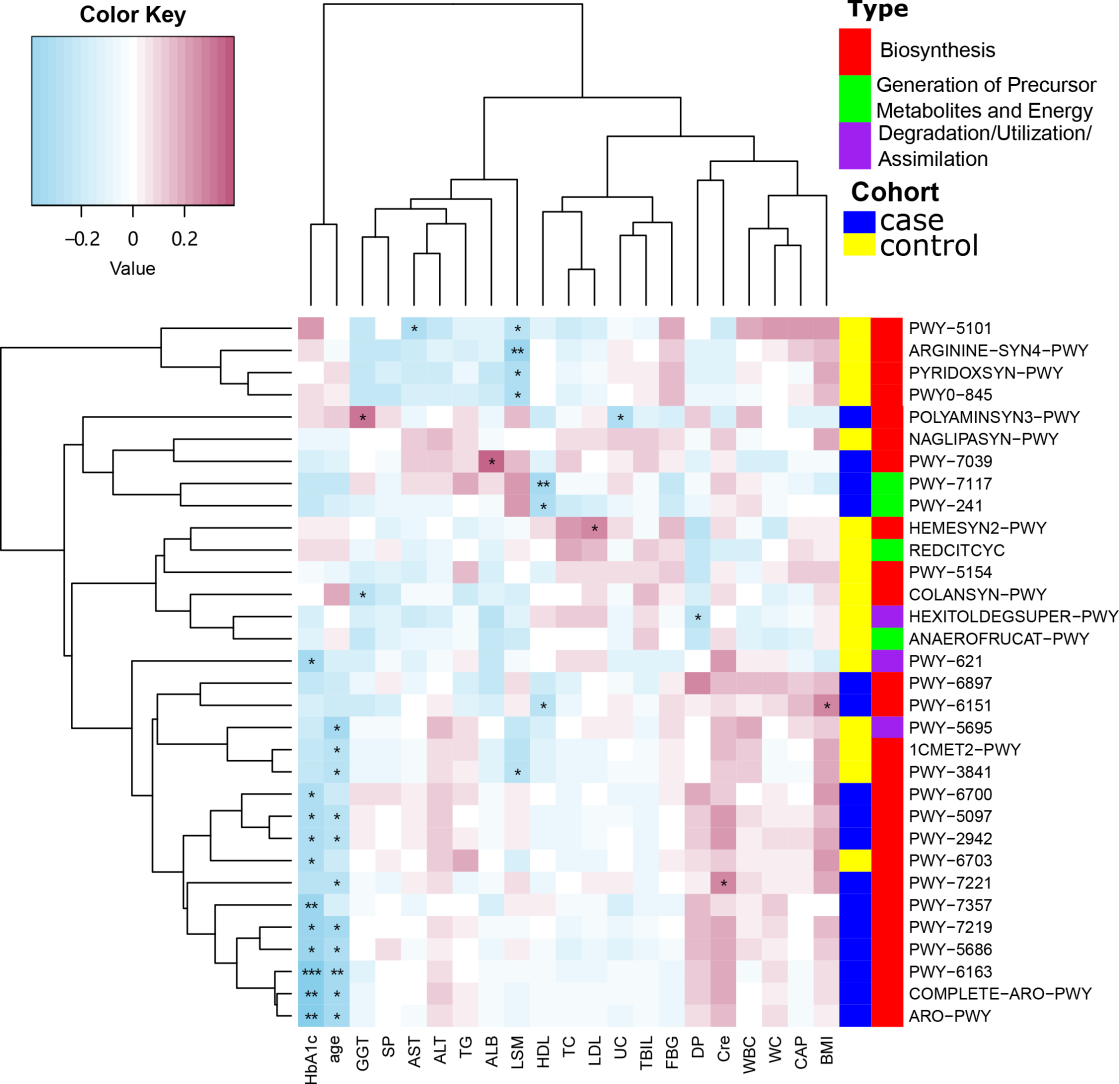
Spearman’s correlation analysis showing the correlations between participant characteristics and MetaCyc pathways. ^*^ *P* < 0.05, ^**^ *P* < 0.01, ^***^ *P* < 0.001.

The correlation between MetaCyc pathways that showed significant differences between the case and control groups and other clinical features was then examined using Spearman’s correlation analysis. The MetaCyc pathways were clustered based on MetaCyc pathway hierarchy and constructed into a heatmap (**Figure 6**). We observed that some pathways were negatively correlated with LSM in the control group, while the majority of pathways in the case group, as well as a subset of pathways in the control group, were negatively correlated with both HbA1c and age.

### Establishment and verification of a predictive model of liver fibrosis by the gut microbiome

**Figure 7:**
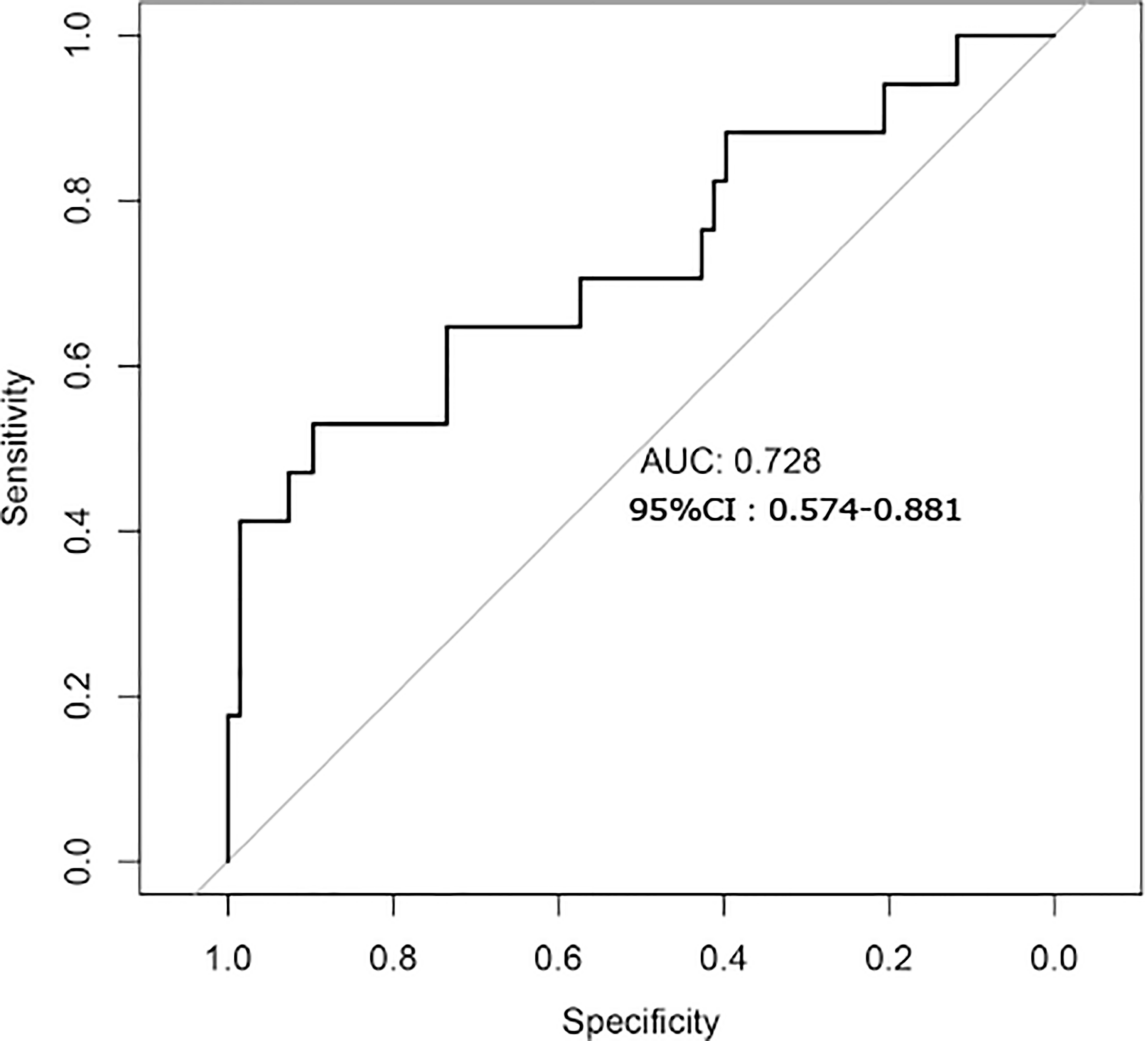
Establishment of a predictive model of liver fibrosis by gut microbiome analysis.

Finally, we used a random forest model to build a predictive model for liver fibrosis (**Figure 7**). The model was constructed using seven bacterial species (*Prevotella copri, Phascolarctobacterium succinatutens, Eubacterium biforme*, *Collinsella aerofaciens, Bacteroides coprocola, Bacteroides stercoris* and *Clostridiales bacterium 1_7_47FAA*). The area under the curve (AUC) for the model was 0.728 (95%; confidence interval: 0.574–0.881). The model showed good diagnostic performance.

## Discussion

While the pathogenesis of MAFLD is not well understood, recent studies have shown that the ecological diversity within the gut microbiota of MAFLD patients is reduced, and that the bacterial composition is altered (Hernández-Ceballos et al., 2021; Oh et al., 2021; Wu et al., 2021). Here, we have characterized and compared the composition and function of the gut microbiota in patients with normal and abnormal LSM scores, and showed that significant differences exist between the two groups.

We observed seven species of bacteria that differed between the case and control groups, belonging to the phyla Bacteroidetes, Firmicutes and Actinobacteria. Both bacterial community richness (measured by the Shannon index and the obs index) and community diversity (measured by the Bray–Curtis test and the Pearson test) showed no significant differences between the case and control groups in our study (*P* > 0.05). At the species level, however, the relative abundances of *Prevotella copri*, *Phascolarctobacterium succinatutens*, *Eubacterium biforme* and *Collinsella aerofaciens* were increased in the case group. Such changes in the microbiota may alter the metabolic pathways occurring in patients’ guts. A previous study observed that the level of *Prevotella copri* in patients with liver cirrhosis was reduced compared with healthy people, which is inconsistent with our data (Zheng et al., 2018). This may be because the patients in our study were in earlier stages of liver fibrosis. Current research suggests that the major mechanisms of intestinal microbiota disorder leading to liver fibrosis are impaired intestinal barrier function, increased permeability of the intestinal wall, and increased production of lipopolysaccharide (Wunsch et al., 2010; Zhang et al., 2019). Bacteria is transferred to the liver through the portal vein, and is then recognized by the TLR4 receptor (Song et al., 2018). This initiates the TLR4/MyD-88/NF-κB pathway, which acts to transcriptionally regulate a number of biological pathways, including stress response, the cell cycle, cellular proliferation, and apoptosis. These pathways ultimately prevent hepatic stellate cells from undergoing apoptosis, which in turn may lead to the development of liver fibrosis (Sawada et al., 2019; Deng et al., 2020).

In this study, we observed that the abundance of *Prevotella copri* was significantly increased in the case group compared with the matched control group. Previous studies found that this increase correlates with elevated concentrations of molecules including lipopolysaccharides, branched-chain amino acids, aromatic amino acids, and arachidonic acid (Chen et al., 2021). High levels of this bacteria can also activate the slow inflammatory response in the liver through the TLR4 signaling pathway, causing liver disease. However, more research is required to fully reveal the relationship between *Prevotella copri* and liver fibrosis.

We also observed other bacterial species that were enriched in the case group for which there is no known link to liver fibrosis. However, the identified species have important biological roles, and so more research could shed light on their roles. *Phascolarctobacterium* produces short-chain fatty acids, including acetate and propionate, and typically uses succinate produced by other bacteria (Wu et al., 2017; Naderpoor et al., 2019; Liu et al., 2020). Notably*, Phascolarctobacterium* was found to be highly enriched in patients with depression, Alzheimer’s disease, autism, and other diseases, while it is reduced in patients with early stage liver cancer (Ogata et al., 2019). *Eubacterium biforme* has been observed to be absent in the intestines of patients with chronic diarrhea (Kišidayová, et al., 2010). *Collinsella aerofaciens* is the most abundant actinomycete in the healthy human gastrointestinal tract. Alterations in its abundance have been suggested to be associated with several health conditions, including irritable bowel syndrome (Lahti et al., 2013; Rajilić-Stojanović et al., 2014).

We then analyzed the metabolic pathways of the bacteria found to be enriched in our case group and observed that the enriched pathways were predominantly involved in the biosynthesis of metabolites, including amino acids, vitamins, nucleosides, and nucleotides. Among these, two pathways (ARO-PWY and PWY-6163) are related to the biosynthesis of aromatic amino acids. A number of studies have shown that when liver fibrosis and cirrhosis occur, amino acid metabolism in the body becomes disordered, and levels of aromatic amino acids are significantly increased (Fukui et al., 2020; Liang et al., 2020; Mizuno et al., 2020). Notably, an increase in aromatic amino acid levels was also associated with an increase in the abundance of *Prevotella* bacteria. In this study, we found that our MALFD case group showed an enrichment of *Prevotella*, and our data also suggested an increase in the ARO-PWY and PWY-6163 aromatic amino acid synthetic pathways. This suggests that the marked changes to the gut microbiota observed during early increases in liver stiffness may lead to a signaling cascade that ultimately alters the metabolic state of the liver. By studying the relationship between changes in gut microbiota and liver stiffness, we can therefore begin to clarify how changes in the gut microbiota may serve as an early sign of liver fibrosis, which may provide a new platform for therapeutic research (Li et al., 2020).

## Conclusions

We found that the structure and function of the gut microbiota changed in the LSM ≥7.4 kpa group. Specifically, *Prevotella* was significantly enriched in the case group. The increased abundance of *Prevotella* was associated with increased concentrations of LPS, branched-chain amino acids, aromatic amino acids, and arachidonic acid metabolites. While for the pathways, ARO-PWY and PWY-6163 involved in aromatic amino acid metabolism were enriched in the case group. Further, gut microbiome analysis was used to establish a predictive model which showed the curve (AUC) for the model was 0.728 (95%; confidence interval: 0.574–0.881). However, the liver fibrosis is not validated by pathologic findings.

## Data availability statement

All sequences have been uploaded into the European Nucleotide Archive (PRJEB48022). Demographic features and serum index measurements used in this study are only available for academic usage.

## Ethics statement

The study was approved by the ethics committee from the First Affiliated Hospital of Zhengzhou University (Number:2018-KY-56 and 2018-KY-90). The patients/participants provided their written informed consent to participate in this study.

## Author contributions

Conceptualization SD and YZ. Methodology and formal analysis: SY and JC. Resources: QQ, WL, SS, TL, XG and LW. Original draft preparation: YZ. Review and editing: SD and LA. Visualization: SY and JC. All authors contributed to the article and approved the submitted version.

## Funding

This research was equally supported and funded by the Henan Province Medical Science and Technology Research Plan (LHGJ20200311, LHGJ20200279), the Chinese National Science and Technology Major Project (2018ZX10305410), the Henan Province Youth Talent Promotion Project (2021HYTP052), and the Henan Province Postdoctoral Research grant (001801005).

## Acknowledgments

The authors thank the group members from Henan Genomics Hospital trial. We also thank the clinicians and the participants who were enrolled in this study. We thank Alison Inglis, PhD, from Liwen Bianji (Edanz) (www.liwenbianji.cn) for editing the English text of a draft of this manuscript.

## Conflict of interest

The authors declare that the research was conducted in the absence of any commercial or financial relationships that could be construed as a potential conflict of interest.

## Publisher’s note

All claims expressed in this article are solely those of the authors and do not necessarily represent those of their affiliated organizations, or those of the publisher, the editors and the reviewers. Any product that may be evaluated in this article, or claim that may be made by its manufacturer, is not guaranteed or endorsed by the publisher.

## Supplementary material

Supplementary Figure 1 Fungi distribution between the two groups.

Supplementary Table 1 The Kolmogorov–Smirnov test was used to test for normality and Levene’s test was used to test the homogeneity of variances.

Supplementary Table 2 The pathways identified by MetaCyc analysis.

## References

[B1] Beraza N. (2019). Fibrosis and the intestinal microbiome: a focus on chronic liver disease. Curr. Opin. Pharmacol. 49, 76–81. doi: 10.1016/j.coph.2019.09.012 31670055

[B2] Campana L. Iredale J. P. (2017). Regression of liver fibrosis. Semin. Liver Dis. 37 (1), 1–10. doi: 10.1055/s-0036-1597816 28201843

[B3] Chen C. Fang S. Wei H. He M. Huang L. (2021). Prevotella copri increases fat accumulation in pigs fed by formula diets. Microbiome 9, 175. doi: 10.1186/s40168-021-01110-0 34419147 PMC8380364

[B4] Chinese Medical Association Liver Disease Branch, Chinese Medical Association Digestive Disease Branch, and Chinese Medical Association Infectious Disease Branch (2019). Diagnosis and treatment consensus of liver fibrosis (2019). Gastrointestinal Dis. 24 (9), 11. doi: 10.3969j.issn.1008-7125.2019.09.007

[B5] Deng Y. Ge S. Yu Y. Xiong Y. (2020). Hepatic fibrosis-related cell signal transduction pathway and potential treatment strategy. J. Clin. Hep Dis. 36 (5), 1141–1145. doi: 10.3969/j.issn.1001-5256.2020.05.043

[B6] Deng H. Wang C. L. Lai J. Yu S. L. Xie D. Y. Gao Z. L. (2016). Noninvasive diagnosis of hepatic steatosis using fat attenuation parameter measured by FibroTouch and a new algorithm in CHB patients. Hepat Mon 16 (9), e40263. doi: 10.5812/hepatmon.40263 27822268 PMC5088638

[B7] Eslam M. Newsome P. N. Anstee Q. M. Targher G. George J. (2020). A new definition for metabolic dysfunction-associated fatty liver disease: An international expert consensus statement. J. Hepatol. 73 (1), 202–209. doi: 10.1016/j.jhep.2020.03.039 32278004

[B8] Franzosa E. A. McIver L. J. Rahnavard G. Thompson L. R. Schirmer M. Weingart G.. (2018). Species-level functional profiling of metagenomes and metatranscriptomes. Nat. Methods 15, 962–968. doi: 10.1038/s41592-018-0176-y 30377376 PMC6235447

[B9] Fukui A. Kawabe N. Hashimoto S. Kamei H. Yoshioka K. (2020). Switching from branched-chain amino acid granules to branched-chain amino acid-enriched nutrient improves the branched-chain amino acid-to-tyrosine ratio in patients with cirrhosis with hypoalbuminemia. Eur. J. Gastroen Hepat 32 (4), 501–506. doi: 10.1097/MEG.0000000000001544 31524770

[B10] Hernández-Ceballos W. Cordova-Gallardo J. Mendez-Sanchez N. (2021). Gut microbiota in metabolic-associated fatty liver disease and in other chronic metabolic diseases[J]. J. Clin. Transl. Hepato 9 (2), 12. doi: 10.14218/JCTH.2020.00131 PMC8111113 34007805

[B11] Kišidayová S. Váradyová Z. Pristaš P. Piknová M. Nigutová K. Petrželková K. J.. (2010). Effects of high- and low-fiber diets on fecal fermentation and fecal microbial populations of captive chimpanzees. Am. J. Primatol 71 (7), 548–557. doi: 10.1002/ajp.20687 19367605

[B12] Kisseleva T. Brenner D. (2021). Molecular and cellular mechanisms of liver fibrosis and its regression. Nat. Rev. Gastro Hepat 18 (3), 151–166. doi: 10.1038/s41575-020-00372-7 33128017

[B13] Lahti L. Salonen A. Kekkonen R. A. Salojärvi J. Jalanka-Tuovinen J. Palva A.. (2013). Associations between the human intestinal microbiota And serum lipids indicated by integrated analysis of high-throughput profiling data. Peer J. 1, e32. doi: 10.7717/peerj.32 23638368 PMC3628737

[B14] Liang K. H. Cheng M. L. Lo C. J. Lin Y. H. Yeh C. T. (2020). Plasma phenylalanine and glutamine concentrations correlate with subsequent hepatocellular carcinoma occurrence in liver cirrhosis patients: an exploratory study. Sci. Rep-UK 10 (1), 10926. 10.1038/s41598-020-67971-x PMC7331577 32616821

[B15] Li Z. Ni M. Yu H. Wang L. Zhou X. Chen T.. (2020). Gut microbiota and liver fibrosis: one potential biomarker for predicting liver fibrosis. BioMed. Res. Int. 2020, 3905130. doi: 10.1155/2020/3905130 32685479 PMC7322594

[B16] Liu S. An Y. Cao B. Sun R. Ke J. Zhao D. (2020). The composition of gut microbiota in patients bearing hashimoto’s thyroiditis with euthyroidism and hypothyroidism. Int. J. Endocrinol. 2020, 9. doi: 10.1155/2020/5036959 PMC7673947 33224194

[B17] Mizuno Y. Ishikawa T. Ishida J. Kobayashi A. Saruta M. (2020). The molar ratio of total branched-chain amino acids to tyrosine predicts a digit symbol test abnormality in cirrhotic patients. Internal Med. 59 (14). doi: 10.2169/internalmedicine.4298-19 PMC7434536 32296001

[B18] Naderpoor N. Mousa A. Gomez-Arango L. Barrett H. Dekker Nitert M. de Courten B. (2019). Faecal microbiota are related to insulin sensitivity and secretion in overweight or obese adults. J. Clin. Med. 8 (4), p. doi: 10.3390/jcm8040452 PMC6518043 30987356

[B19] Ogata Y. Suda W. Ikeyama N. Hattori M. Ohkuma M. Sakamoto M. (2019). Complete genome sequence of phascolarctobacterium faecium jcm 30894, a succinate-utilizing bacterium isolated from human feces. Microbiol. Resour Ann. 8 (3), e01487–e01418. doi: 10.1128/MRA.01487-18 PMC6346166 30687834

[B20] Oh J. H. Lee J. H. Min S. C. Kim H. Kang W. (2021). Characterization of gut microbiome in korean patients with metabolic associated fatty liver disease. Nutrients 13, 1013. doi: 10.3390/nu13031013 33801023 PMC8004024

[B21] Qin T. Fu J. Verkade H. J. (2021). The role of the gut microbiome in graft fibrosis after pediatric liver transplantation. Hum. Genet. 140 (5), 709–724. doi: 10.1007/s00439-020-02221-8 32920649 PMC8052232

[B22] Qu Y. Song Y. Y. Chen C. W. Fu Q. C. Shi J. P. Xu Y.. (2021). Diagnostic performance of FibroTouch ultrasound attenuation parameter and liver stiffness measurement in assessing hepatic steatosis and fibrosis in patients with nonalcoholic fatty liver disease. Clin. Transl. Gastroen 12 (4), e00323. doi: 10.14309/ctg.0000000000000323 PMC8049161 33848277

[B23] Rajilić-Stojanović M. De Vos W. M. (2014). The first 1000 cultured species of the human gastrointestinal microbiota. FEMS Microbiol. Rev. 38, 996–1047. doi: 10.1111/1574-6976.12075 24861948 PMC4262072

[B24] Sawada Y. Kawaratani H. Kubo T. Fujinaga Y. Yoshiji H. (2019). Combining probiotics and an angiotensin-II type 1 receptor blocker has beneficial effects on hepatic fibrogenesis in a rat model of non-alcoholic steatohepatitis. Hepatol. Res. 49 (3), 284–295. doi: 10.1111/hepr.13281 30365236

[B25] Song I. J. Yang Y. M. Inokuchi-Shimizu S. Roh Y. S. Yang L. Ekihiro S. (2018). The contribution of toll-like receptor signaling to the development of liver fibrosis and cancer in hepatocyte-specific TAK1-deleted mice. Int. J. Cancer 142 (1), 81–91. doi: 10.1002/ijc.31029 28875549 PMC5790193

[B26] Sun M. Kisseleva T. (2015). Reversibility of liver fibrosis. Clin. Res. Hepatol. Gas 39 Suppl 1 (01), S60–S63. doi: 10.1016/j.clinre.2015.06.015 PMC4636085 26206574

[B27] Tilg H. Cani P. D. Mayer E. A. (2016). Gut microbiome and liver diseases. Gut 65 (12), 2035. doi: 10.1136/gutjnl-2016-312729 27802157

[B28] Truong D. T. Franzosa E. A. Tickle T. L. Scholz M. Weingart G. Pasolli E.. (2015). MetaPhlAn2 for enhanced metagenomic taxonomic profiling. Nat. Methods 12, 902–903. doi: 10.1038/nmeth.3589 26418763

[B29] Wu W. K. Chen Y. H. Lee P. C. Yang P. J. Wu M. S. (2021). Mining gut microbiota from bariatric surgery for MAFLD. Front. Endocrinol. (Lausanne) 12, 612946. doi: 10.3389/fendo.2021.612946 33897617 PMC8063105

[B30] Wu F. Guo X. Zhang J. Zhang M. Ou Z. Peng Y.. (2017). Phascolarctobacterium faecium abundant colonization in human gastrointestinal tract. Exp. Ther. Med. 14 (4), 3122–3126. doi: 10.3892/etm.2017.4878 28912861 PMC5585883

[B31] Wunsch E. Marlicz W. Milkiewicz P. (2010). Probiotics in chronic liver diseases. Pol. Merkur Lekarski 29 (174), 390–394. 21298992

[B32] Xu L. Lu W. Li P. Shen F. Mi Y. Q. Fan J. G. (2017). A comparison of hepatic steatosis index, controlled attenuation parameter and ultrasound as noninvasive diagnostic tools for steatosis in chronic hepatitis b. Digest Liver Dis. 49 (8), 910–917. doi: 10.1016/j.dld.2017.03.013 28433586

[B33] Zhang L. Wu Y. N. Chen T. Ren C. H. Li X. Liu G. X. (2019). Relationship between intestinal microbial dysbiosis and primary liver cancer. Hepatob Pancreat Dis. 18 (2), 149–157. doi: 10.1016/j.hbpd.2019.01.002 30661942

[B34] Zheng T. Jiang S. Fang Z.. (2018). Study on the change of intestinal strength of patients with chronic hepatitis b, cirrhosis and liver failure. Pract. Liver Dis. 21 (6), 899–902. doi: 10.3969/j.issn.1672-5069.2018.06.018

[B35] Zhou R. Fan X. Schnabl B. (2019). Role of the intestinal microbiome in liver fibrosis development and new treatment strategies. Transl. Res. 209, 22–38. doi: 10.1016/j.trsl.2019.02.005 30853445

[B36] Zhu S. H. Zheng K. I. Hu D. S. Gao F. Zheng M. H. (2021). Optimal thresholds for ultrasound attenuation parameter in the evaluation of hepatic steatosis severity: evidence from a cohort of patients with biopsy-proven fatty liver disease. Eur. J. Gastroen Hepat 33 (3), 430–435. doi: 10.1097/MEG.0000000000001746 32398489

