# Supplementary material for: Comparison of gut microbiota in male MAFLD patients with varying liver stiffness
Source: Front Cell Infect Microbiol. 2022 Aug 3;12:873048. doi: 10.3389/fcimb.2022.873048 (PMC9381746; doi:10.3389/fcimb.2022.873048)
Supplement: Supplementary Figure 1 — Fungi distribution between the two groups. [file DataSheet_1.docx]

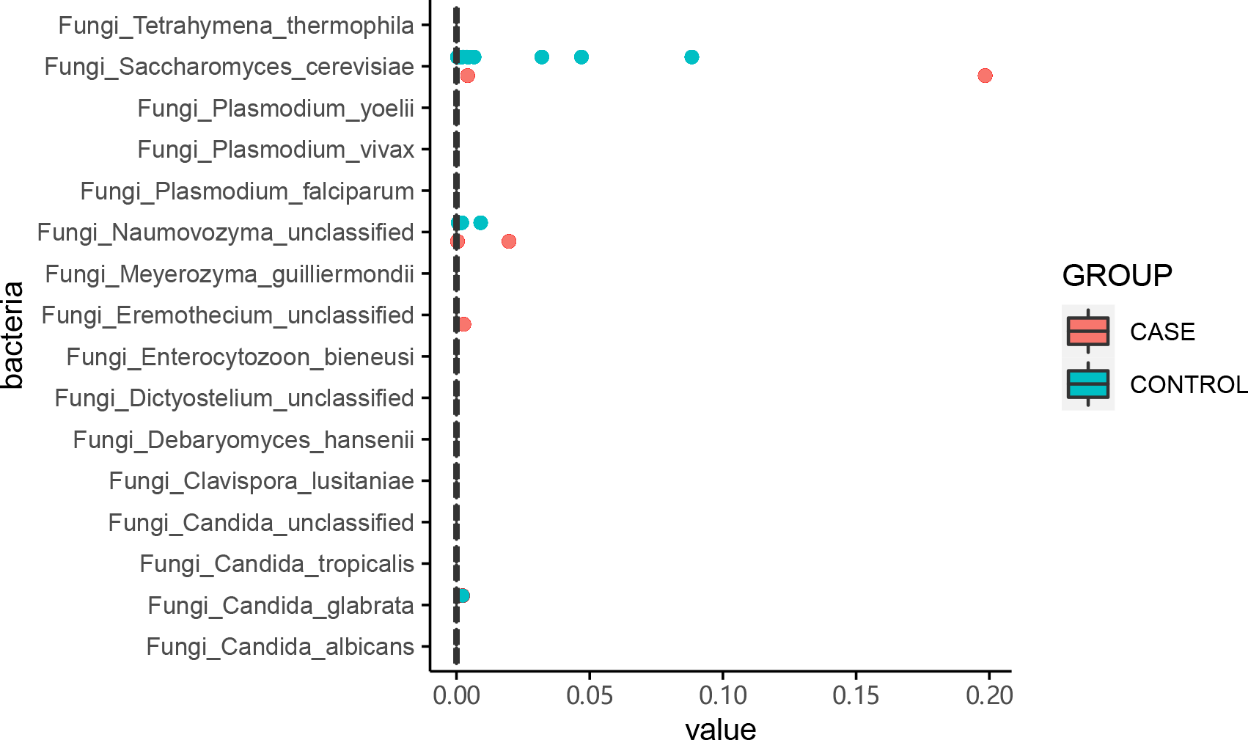


**Supplementary Figure 1. Fungi distribution between the two groups.**

**Supplementary Table 1. The Kolmogorov–Smirnov test was used to test for normality and Levene’s test was used to test the homogeneity of variances.**

|  | | control | | case | | levene | |
| --- | --- | --- | --- | --- | --- | --- | --- |
|  | Kolmogorov-Smirnov | | | Kolmogorov-Smirnov | |  |  |
|  | D | | p-value | D | p-value | F | p-value |
| Age | 0.076 | | 0.2 | 0.133 | 0.2 | 0.395 | 0.531 |
| WC(cm) | 0.074 | | 0.2 | 0.102 | 0.2 | 0.596 | 0.442 |
| SBP(mmHg) | 0.062 | | 0.2 | 0.203 | 0.06 | 0.24 | 0.625 |
| DBP(mmHg) | 0.058 | | 0.2 | 0.118 | 0.2 | 0.005 | 0.944 |
| BMI(kg/m^2^) | 0.145 | | 0.001 | 0.184 | 0.129 | 0.009 | 0.925 |
| WBC(×10^9^/L) | 0.08 | | 0.2 | 0.123 | 0.2 | 3.307 | 0.073 |
| ALT(U/L) | 0.104 | | 0.066 | 0.313 | <0.001 | 32.511 | <0.001 |
| AST(U/L) | 0.13 | | 0.006 | 0.368 | <0.001 | 25.836 | <0.001 |
| GGT(U/L) | 0.186 | | <0.001 | 0.248 | 0.007 | 2.368 | 0.128 |
| ALB(g/L) | 0.061 | | 0.2 | 0.16 | 0.2 | 0.499 | 0.482 |
| TBIL(μmol/L) | 0.144 | | 0.001 | 0.146 | 0.2 | 3.083 | 0.083 |
| Cr(μmol/L) | 0.109 | | 0.045 | 0.207 | 0.051 | 0.278 | 0.599 |
| SUA(μmol/L) | 0.084 | | 0.2 | 0.098 | 0.2 | 0.469 | 0.495 |
| TC(mmol/L) | 0.11 | | 0.039 | 0.113 | 0.2 | 0.572 | 0.452 |
| TG(mmol/L) | 0.162 | | <0.001 | 0.181 | 0.142 | 0.316 | 0.575 |
| HDL(mmol/L) | 0.098 | | 0.18 | 0.12 | 0.2 | 0.018 | 0.894 |
| LDL(mmol/L) | 0.072 | | 0.2 | 0.153 | 0.2 | 0.032 | 0.858 |
| FPG(mmol/L) | 0.193 | | <0.001 | 0.251 | 0.006 | 0.979 | 0.325 |
| HbA1c(%) | 0.239 | | <0.001 | 0.207 | 0.165 | 2.763 | 0.101 |
| CAP(dB/m) | 0.149 | | 0.001 | 0.123 | 0.2 | 0.467 | 0.496 |
| LSM(kPa) | 0.071 | | 0.2 | 0.173 | 0.184 | 1.038 | 0.311 |

**Supplementary Table 2. The pathways identified by MetaCyc analysis.**

| ID | name |
| --- | --- |
| PWY-6897 | thiamin_salvage_II |
| PWY-5686 | UMP_biosynthesis |
| POLYAMINSYN3-PWY | superpathway_of_polyamine_biosynthesis_II |
| PWY-7221 | guanosine_ribonucleotides_de_novo_biosynthesis |
| HEXITOLDEGSUPER-PWY | superpathway_of_hexitol_degradation_(bacteria) |
| 1CMET2-PWY | N10-formyl-tetrahydrofolate_biosynthesis |
| PWY-5097 | L-lysine_biosynthesis_VI |
| PWY-6163 | chorismate_biosynthesis_from_3-dehydroquinate |
| PYRIDOXSYN-PWY | pyridoxal_5'-phosphate_biosynthesis_I |
| ARO-PWY | chorismate_biosynthesis_I |
| PWY-6700 | queuosine_biosynthesis |
| PWY-5101 | L-isoleucine_biosynthesis_II |
| PWY-7117 | C4_photosynthetic_carbon_assimilation_cycle,_PEPCK_type |
| PWY-2942 | L-lysine_biosynthesis_III |
| PWY-7357 | thiamin_formation_from_pyrithiamine_and_oxythiamine_(yeast) |
| NAGLIPASYN-PWY | lipid_IVA_biosynthesis |
| PWY-7219 | adenosine_ribonucleotides_de_novo_biosynthesis |
| COMPLETE-ARO-PWY | superpathway_of_aromatic_amino_acid_biosynthesis |
| COLANSYN-PWY | colanic_acid_building_blocks_biosynthesis |
| PWY-3841 | folate_transformations_II |
| ARGININE-SYN4-PWY | L-ornithine_de_novo__biosynthesis |
| PWY-6703 | preQ0_biosynthesis |
| HEMESYN2-PWY | heme_biosynthesis_II_(anaerobic) |
| PWY-5695 | urate_biosynthesis/inosine_5'-phosphate_degradation |
| ANAEROFRUCAT-PWY | homolactic_fermentation |
| PWY-6151 | S-adenosyl-L-methionine_cycle_I |
| PWY0-845 | superpathway_of_pyridoxal_5'-phosphate_biosynthesis_and_salvage |
| PWY-5154 | L-arginine_biosynthesis_III_(via_N-acetyl-L-citrulline) |
| PWY-241 | C4_photosynthetic_carbon_assimilation_cycle,_NADP-ME_type |
| REDCITCYC | TCA_cycle_VIII_(helicobacter) |
| PWY-621 | sucrose_degradation_III_(sucrose_invertase) |
| PWY-7039 | phosphatidate_metabolism,_as_a_signaling_molecule |
